# Effect of Myofascial Release on Pain and Uterine Artery Hemodynamic Indices in Women with Primary Dysmenorrhea: A Randomized Controlled Trial

**DOI:** 10.3390/medicina61101736

**Published:** 2025-09-24

**Authors:** Shiyu Jin, Jongwon Choi, Haneul Lee

**Affiliations:** 1 Graduate School, Department of Physical Therapy, College of Medical Science, Gachon University, 191 Hambakmae-ro, Yeonsu-gu, Incheon 21936, Republic of Korea; kimseok17@gachon.ac.kr (S.J.); mfrkorea@gmail.com (J.C.); 2 Department of Physical Therapy, College of Medical Science, Gachon University, 191 Hambakmae-ro, Yeonsu-gu, Incheon 21936, Republic of Korea

**Keywords:** myofascial release, non-pharmacological intervention, primary dysmenorrhea, uterine artery hemodynamics

## Abstract

***Background***: Primary dysmenorrhea (PD) is a common gynecological condition among women of reproductive age, often leading to pain and functional limitations. Myofascial release (MFR) has been suggested as a potential non-pharmacological intervention. This study aimed to investigate the immediate effects of a single MFR session on pain intensity, menstrual symptoms, and uterine artery hemodynamics in women with PD. ***Methods***: In this randomized controlled trial, 34 women with PD were randomly assigned to either the MFR group (*n* = 18) or the placebo MFR group (*n* = 16). All participants received 10 min of thermotherapy followed by 30 min of either MFR or placebo MFR. Pain intensity (NRS), pressure pain thresholds (PPT) at myofascial trigger points, menstrual symptoms (MDQ-T), and uterine artery pulsatility index (PI) and resistance index (RI) were assessed at three time points: baseline, immediately after the intervention, and 3 h post-intervention. ***Results***: Both groups demonstrated significant within-group reductions in pain intensity and menstrual symptoms post-intervention (*p* < 0.01), with no significant group-by-time interaction. However, significant interaction effects were observed for the PI and RI of the right uterine artery, showing greater reductions in the MFR group compared to the placebo group at 3 h post-intervention (*p* < 0.05). ***Conclusions***: A single MFR session resulted in improvements in uterine hemodynamics, suggesting autonomic modulation as a potential mechanism. Although subjective symptom improvements were observed in both groups, only MFR showed objective vascular benefits. These findings support the physiological plausibility of MFR in PD management and suggest its potential application as a personalized, non-pharmacological intervention. Further studies are warranted to explore its long-term and individualized therapeutic effects.

## 1. Introduction

Primary dysmenorrhea (PD) is one of the most prevalent gynecological conditions, defined as recurrent cramping pain in the lower abdomen that occurs immediately before or during menstruation in the absence of identifiable pelvic pathology [1]. It is a leading cause of chronic and cyclic pelvic pain among women of reproductive age. Globally, more than 50% of menstruating women experience some degree of menstrual pain, and over 90% of adolescents report such symptoms, with 10% to 20% suffering from acute or debilitating pain that interferes with daily activities [2]. Despite the high incidence, PD is frequently underdiagnosed and undertreated, particularly in young women, who tend to perceive menstrual pain as a normal part of menstruation rather than a medical concern.

The pathophysiology of PD involves complex neurovascular and endocrine mechanisms [3]. The pain typically presents as sharp, cramping sensations in the suprapubic region and may radiate to the lower back or inner thighs [4]. The most intense discomfort usually occurs within the first 24 to 36 h after the onset of menstruation, coinciding with the peak release of prostaglandins from the endometrium [5]. In particular, excessive secretion of prostaglandin F2α (PGF2α) leads to exaggerated uterine contractions, elevated intrauterine pressure, and reduced uterine perfusion, causing hypoxia and ischemic pain [3,6]. Additionally, vasopressin-mediated vasoconstriction further compromises pelvic blood flow and contributes to metabolic dysfunction and myofascial hypersensitivity in the pelvic and abdominal musculature [7,8]. Such hemodynamic disturbances exacerbate uterine ischemia, contribute to nociceptor activation, and have been positively correlated with the severity of menstrual symptoms [9,10]. These peripheral mechanisms may be compounded by autonomic imbalance and central sensitization of pain pathways [11,12].

Pharmacologic interventions such as nonsteroidal anti-inflammatory drugs (NSAIDs) and hormonal contraceptives have been widely used, offering effective symptom relief. However, long-term medication use can have adverse effects and may not be suitable for all patients [13]. As a result, there is growing interest in complementary and non-pharmacologic approaches [14,15]. Interventions such as isometric exercise, massage therapy, connective tissue manipulation, stretching, Kinesio taping, and progressive relaxation have shown promise in alleviating menstrual pain without systemic side effects [16].

Among these interventions, myofascial release (MFR) has emerged as a promising manual therapy that targets fascial restrictions to reduce pain and improve tissue mobility [17]. MFR involves the application of gentle, sustained pressure to stretch and mobilize myofascial tissues, thereby alleviating tension, improving circulation, and restoring muscular and fascial balance [11,18,19]. Recent clinical studies have demonstrated that MFR may reduce menstrual pain and decrease reliance on analgesic medications in women with PD [20]. Moreover, MFR has been shown to increase pressure pain thresholds and reduce fatigue, performing comparably or better than connective tissue massage in women with dysmenorrhea [21]. Mechanistically, MFR stimulates fibroblasts within the fascial matrix, promoting the reorganization of collagen and elastin fibers, enhancing interlayer sliding, and restoring fascial elasticity [22]. These changes improve local circulation and facilitate venous and lymphatic drainage, which may contribute to waste product removal, edema reduction, and tissue recovery. A recent review further emphasized that MFR may alleviate fascial compression on vascular structures, thereby improving tissue oxygenation and blood flow [23]. Collectively, these findings suggest that MFR elicits not only structural but also physiological responses that may benefit women suffering from PD.

The therapeutic effects of MFR on musculoskeletal and visceral pain are increasingly supported by evidence [24,25]. However, the effects of a single-session MFR intervention on menstrual pain and uterine artery hemodynamics remain underexplored. Considering the multifactorial nature of PD, which involves both neurovascular dysregulation and myofascial involvement, a more comprehensive evaluation incorporating both subjective and objective measures is warranted to elucidate the full clinical potential of MFR. Therefore, the present study aimed to investigate the effects of a single-session MFR intervention on pain intensity, menstrual symptoms, and uterine artery hemodynamics in women with PD. Building on previous studies, this design uniquely integrates clinical outcomes with physiological measures of uterine blood flow, offering a more comprehensive understanding of the therapeutic effects of MFR. We hypothesized that MFR would produce greater improvements in pain, symptom severity, and uterine artery hemodynamics compared to placebo control.

## 2. Methods

### 2.1. Study Design and Ethical Approval

This randomized controlled trial was conducted in accordance with the Consolidated Standards of Reporting Trials (CONSORT) guidelines and was registered at the Clinical Research Information Service (CRIS) of the Korea National Institute of Health (registration number: KCT0010339; registration date: 26 March 2025). The study protocol was approved by the Institutional Review Board (IRB) of Gachon University (1044396-202501-HR-010-01; approval date: 25 February 2025) and complied with the ethical principles outlined in the Declaration of Helsinki. Prior to participation, all participants were fully informed of the study’s purpose, procedures, and data privacy policies. Written informed consent was obtained from all individuals who voluntarily agreed to participate.

### 2.2. Participants

Sixty-two women with regular menstrual cycles and a clinical history of PD were initially screened for eligibility. Of these, 34 women aged 18–35 years [7,20] met the inclusion criteria and were enrolled in the study. Participants were recruited from a university in Incheon, Republic of Korea, following an approved recruitment procedure. Poster advertisements displayed on official campus bulletin boards to invite eligible participants. Eligible participants were those who (1) were between 18 and 35 years of age; (2) had regular menstrual cycles over the past 12 months (cycle length of 24–35 days); (3) had experienced menstrual pain for at least the past three months; and (4) reported a pain intensity of ≥3 on the Numerical Rating Scale (NRS) during menstruation within the past three months. Women were excluded if they (1) had a body mass index (BMI) ≥30 kg/m^2^; (2) were currently using hormonal contraceptives or intrauterine devices; (3) had a diagnosis of fibromyalgia or other rheumatic conditions; (4) had a history of surgery or fracture involving the lumbar spine, pelvis, or hip joints; or (5) had any known gynecological disorders.

**Figure 1:**
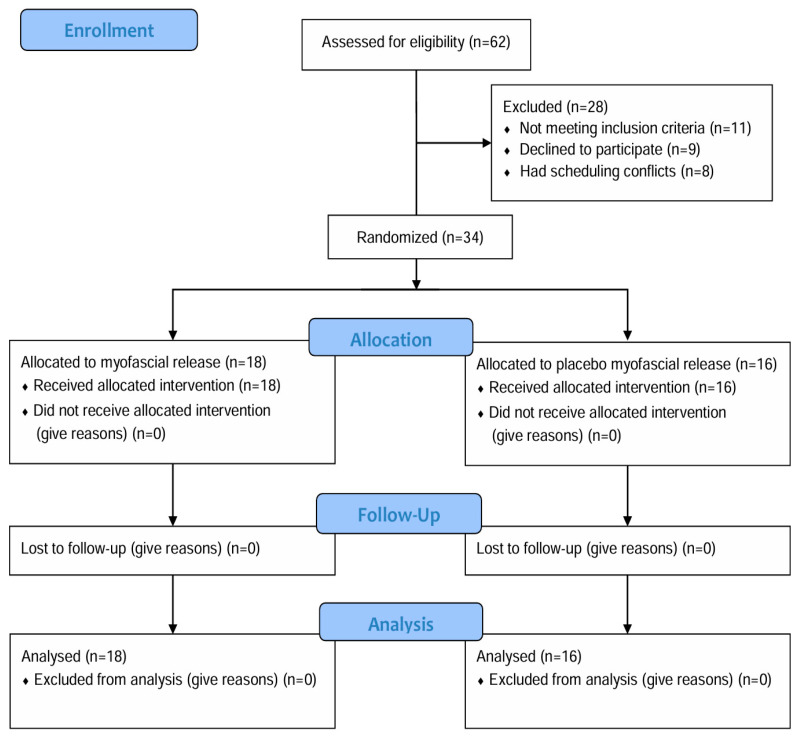
Flow diagram of the study.

A total of 34 participants were randomly assigned to either the MFR (*n* = 18) or placebo MFR group (*n* = 16) using a simple randomization method (Figure 1). Group allocation was performed via a manual lottery procedure, in which identically sized, opaque balls labeled with group names were placed in a sealed, non-transparent container. Each participant drew one ball to determine group assignment. To further ensure allocation concealment, the lottery procedure was supervised by a research assistant not involved in outcome assessment. This method ensured allocation concealment, as neither participants nor the allocating researcher could predict the assignment sequence. The study employed a single-blind design, in which participants were blinded to their group allocation and unaware of whether they received the active MFR intervention or the placebo treatment.

The required sample size was calculated using G*Power software (version 3.1.9.7; Heinrich-Heine-University Düsseldorf, Germany). Based on a two-tailed *t*-test for differences between two dependent means, as pain intensity was measured repeatedly within the same participants across menstrual cycles, the alpha level (α) was set at 0.05, with a statistical power (1−β) of 0.80 and a large expected effect size (Cohen’s *d*) of 1.1 calculated using the mean and standard deviation (SD) values of pain intensity reported in a previous study (MFR group: 4.1 ± 1.48; control group: 5.92 ± 1.81) [20]. The calculation indicated that a minimum of 30 participants would be required to detect a statistically significant difference. To account for potential dropouts and ensure adequate statistical power, a total of 34 participants were recruited for this study.

### 2.3. Procedure

All participants were instructed to refrain from using analgesics starting one day prior to the intervention. A single treatment session was administered on days 2–3 of the menstrual cycle, which typically corresponds to the peak of menstrual pain. Both groups received 10 min of thermotherapy, followed by a 30 min session of either MFR or placebo MFR. To evaluate the effects of the intervention, outcome measures were assessed at three time points: before the intervention (pre), immediately after (post 1), and 3 h post-intervention (post 2). Baseline demographic and menstrual characteristics were assessed prior to the intervention. At each time point, the following outcomes were measured: pain intensity, pressure pain thresholds of myofascial trigger points, menstrual symptoms, and uterine artery hemodynamic indices.

### 2.4. Outcome Measures

#### 2.4.1. Primary Outcome-Menstrual Pain

Menstrual pain intensity was assessed using the 11-point NRS, ranging from 0 (no pain) to 10 (worst imaginable pain). Scores of 1–3 indicate mild pain, 4–7 moderate pain, and 8–10 severe pain. Higher scores indicate greater pain severity. The NRS has demonstrated high reliability for assessing menstrual pain in women, with an intra-class correlation coefficient (ICC) of 0.90 [26]. The minimal clinically important difference (MCID) for MFR-related menstrual pain reduction was defined as 1.65 points [27].

#### 2.4.2. Secondary Outcomes

##### Pressure Pain Threshold at Myofascial Trigger Points

The pressure pain threshold (PPT) was measured at myofascial trigger points using a handheld digital algometer (microFET2, Hoggan Scientific, Salt Lake City, UT, USA) [21]. PPT was measured bilaterally at six sites in the lower abdomen: (1) the pubic symphysis, (2) the intersection between the ASIS and the lateral margin of the rectus abdominis, and (3) approximately 2 cm medial to the ASIS. In the lower back, the measurement site was the sacral region between S2 and S4 vertebral levels. The investigator applied perpendicular pressure to the skin surface using the algometer, and the pressure value at which the participant first perceived pain was recorded as the PPT. Each site was consistently measured three times, and the average value was used for analysis. Higher PPT values indicated lower sensitivity to pressure-induced pain. The reliability of PPT assessment with the microFET2 algometer has been well established, with intraclass correlation coefficients reported to range from 0.63 to 0.98 [28].

##### Menstrual Symptom

Menstrual symptoms were assessed using the Korean version of the Menstrual Distress Questionnaire Form-T (MDQ-T), a self-reported instrument consisting of 47 items across nine subscales: Pain, Water Retention, Autonomic Reactions, Negative Affect, Impaired Concentration, Behavioral Change, Arousal, Control, and Menstrual Flow [29,30]. Each item was rated on a 5-point Likert scale ranging from 0 (not at all) to 4 (severe), with higher total scores indicating greater menstrual symptom severity. The instrument was used under a licensed agreement with the copyright holder, Mind Garden Inc. (Menlo Park, CA, USA). (www.mindgarden.com). The Korean version of the MDQ-T has demonstrated excellent internal consistency, with a reported Cronbach’s alpha of 0.96 [29].

##### Uterine Artery Hemodynamic Indices

Uterine artery hemodynamic parameters were assessed using the RS85 Prestige ultrasound system (Samsung Medison, Seoul, Republic of Korea) equipped with a curved array transducer (CA1-7A, Samsung Medison) operating at a frequency bandwidth of 1.0–7.0 MHz. For uterine artery blood flow measurements, the pulse repetition frequency was set at 2.5 kHz (−42.7 to 42.7 cm/s) in color Doppler mode and 5.0 kHz (−50 to 50 cm/s) in pulsed Doppler mode, with the gain set at 50. Color Doppler imaging was applied to identify the uterine arteries wile pulsed Doppler was used to obtain the pulsatility index (PI) and resistive index (RI). PI and RI were calculated according to Equations (1) and (2) and served as indicators of flow pulsatility and vascular resistance.

(1) $$PI=\frac{peak\;systolic\;velocities\;\left(PSV\right)\;-\;end\;diastolic\;velocities\;(EDV)\;}{mean\;velocity\;(MV)}$$

(2) $$RI=\frac{PSV-EDV}{PSV}$$

**Figure 2:**
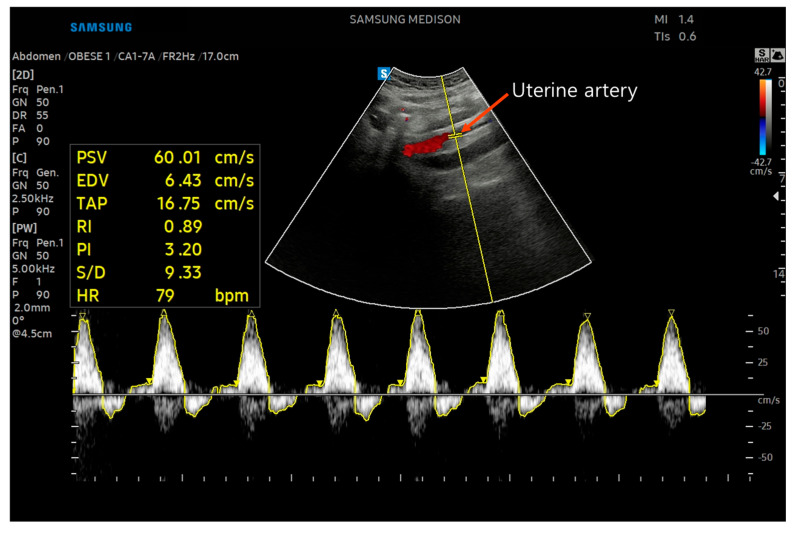
Doppler ultrasound measurement of uterine artery pulsatility index, resistive index.

To visualize the target arteries, participants were placed in the supine position, and the transducer was first placed in transverse orientation just above the pubic symphysis to identify the bladder [31], which served as an anatomical landmark for subsequent vessel localization. The uterine artery, located laterally to the cervix, was located by gradually moving the probe laterally and adjusting to a longitudinal orientation [32]. The arcuate artery, located within the myometrium and branching from the uterine artery, was identified by moving the probe cranially and slightly laterally from the lower abdomen, just below the umbilicus [9] (Figure 2). During measurements, the insonation angle between the Doppler beam and blood flow was maintained below 30° to ensure measurement accuracy, in line with standard Doppler examination practice [32]. Arteries were confirmed when the PSV exceeded 60 cm/s and at least three consecutive consistent waveforms were observed. Each artery was measured three times, and the mean value was used for analysis.

Previous studies have demonstrated high reliability of Doppler ultrasound for uterine blood flow assessment, with ICC of 0.92 for experienced examiners and >0.80 for less experienced ones [33]. In addition, other studies have also reported good reproducibility of uterine artery Doppler measurements [34].

### 2.5. Intervention

#### 2.5.1. Thermotherapy

Thermotherapy was administered for 10 min using two electric hot packs, each measuring 25 × 35 cm, manufactured by NES-100 (SuniHome Co., Ltd., Busan, Republic of Korea), while the participant was in a supine position. One pack was placed on the lower abdomen and the other on the lower back, with temperatures set at 50 °C and 45 °C, respectively. The devices were equipped with built-in thermostatic controls to ensure consistent heat delivery throughout the application.

#### 2.5.2. Myofascial Release

**Figure 3:**
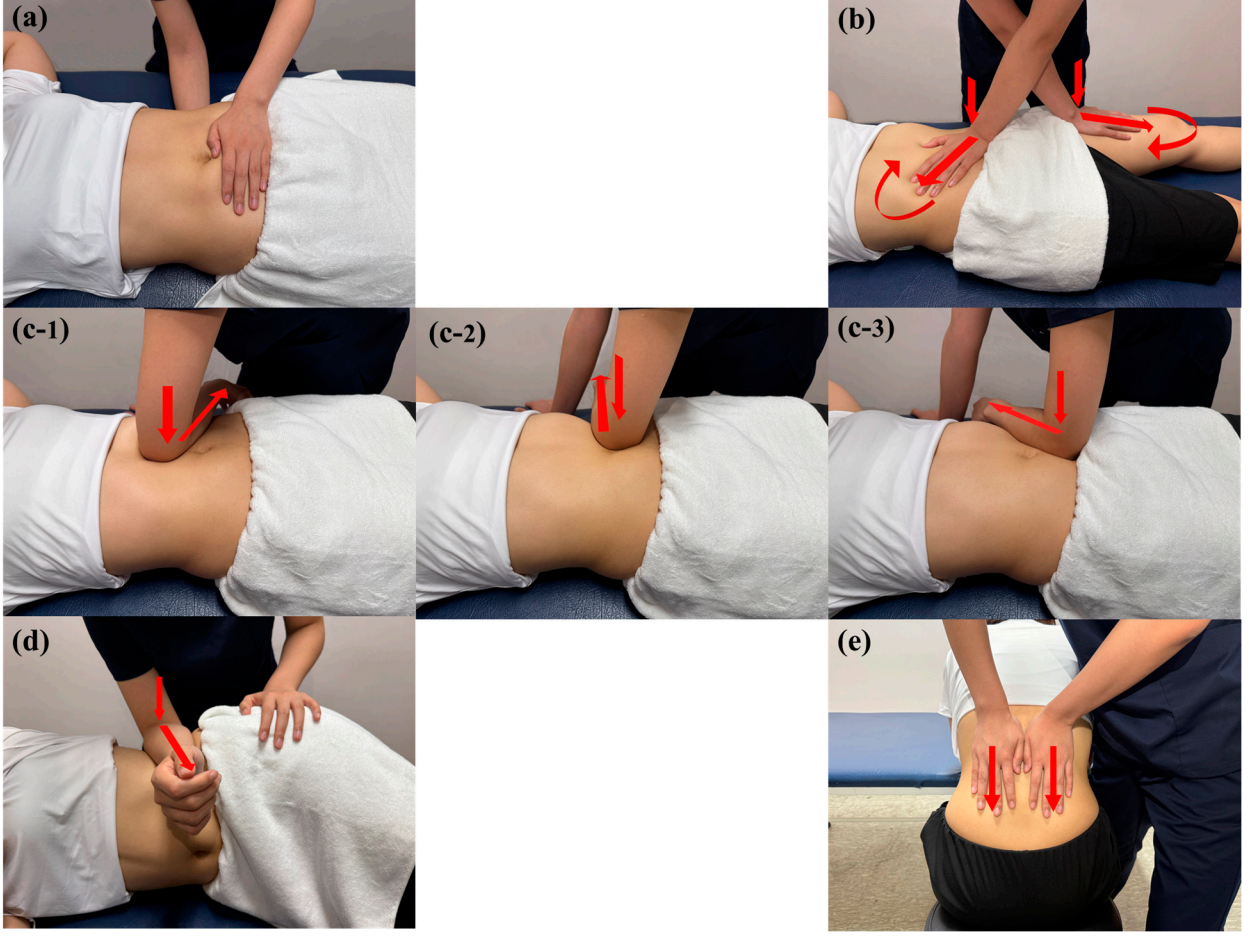
Myofascial release techniques applied to the (**a**) pelvic diaphragm; (**b**) anterior hip; (**c**) abdominal fascia; (**d**) quadratus lumborum fascia; (**e**) thoracolumbar fascia. Red arrows indicate the direction of force applied by the therapist.

Prior to intervention, the therapist warmed their hands to body temperature to ensure comfortable contact. A standardized 30 min MFR protocol was administered using gentle, sustained pressure, longitudinal fascial traction, and tissue twisting, which were maintained until a perceptible release or physiological response such as pain relief, pulling sensation, or tissue softening was achieved. While the overall sequence and techniques were standardized, the pressure and duration at each site were minimally adjusted based on the participant’s tissue response and comfort level to optimize effectiveness. Five techniques were sequentially applied (Figure 3):

(1) Pelvic diaphragm release was applied in the supine position using bilateral hand contact, with one hand placed on the sacral region and the other on the lower abdomen between the ASIS. Anteroposterior compression was applied targeting superficial and deep abdominal fasciae.
(2) Anterior hip release was applied in the supine position using a cross-hand technique, with one hand on the rectus femoris and the other between the ASIS and umbilicus. Gentle pressure was followed by longitudinal traction toward the inguinal region and sustained tissue twisting until a release was perceived.
(3) Abdominal fascia release was applied in the supine position using elbow contact on the anterolateral abdominal wall. Laterally directed pressure was applied in three regions (upper, middle, and lower abdomen), followed by deep fascial mobilization within the pelvic cavity until a release was perceived.
(4) Quadratus lumborum fascia release was applied in the side-lying position using flat elbow pressure. Anteroposterior mobilization was followed by gentle oscillation in the same direction until a release was perceived.
(5) Thoracolumbar fascia release was applied in the seated, forward-flexed position using bilateral hand contact. Sustained downward pressure was applied to the thoracolumbar junction, followed by gradual tension release until a release was perceived.

All techniques targeted key fascial layers, including the superficial fascia, transversalis fascia, extraperitoneal fascia, thoracolumbar fascia, and fasciae surrounding the rectus femoris and quadratus lumborum, to restore fascial mobility across the abdominal–pelvic–lower limb myofascial continuum [20,21].

#### 2.5.3. Placebo Myofascial Release

The placebo MFR intervention was administered for 30 min using the same positioning, duration, and anatomical regions as the active MFR intervention. Therapists were trained to maintain only superficial hand contact and to avoid applying pressure to deeper tissues. Accordingly, the placebo technique was delivered with minimal pressure, ensuring that no mechanical stimulation reached the fascial or muscular layers.

### 2.6. Statistical Analysis

All statistical analyses were performed using IBM SPSS Statistics for Windows, version 28.0 (IBM Corp., Armonk, NY, USA) following the intention-to-treat principle. Normality was assessed using the Kolmogorov–Smirnov test, and homogeneity of variance using Levene’s test. Continuous variables are expressed as means and SD, and categorical variables as frequencies and percentages.

A linear mixed model (LMM) was employed to examine the main effects of time, group (MFR vs. placebo MFR), and their interaction. Age, BMI, regular exercise, menstrual flow, and baseline pain intensity were included as covariates. Bonferroni-adjusted pairwise comparisons were conducted when significant effects were observed. The statistical significance level was set at α = 0.05. For all analyses, within-group effect sizes (Cohen’s *d*) were calculated for pairwise comparisons, and thresholds of 0.1, 0.4, and 0.8 were used to indicate small, medium, and large effects, respectively [35].

## 3. Results

All 34 participants completed the study without any dropouts. Baseline demographic and clinical characteristics did not differ significantly between the MFR and placebo MFR groups. Detailed characteristics are presented in Table 1.

### 3.1. Menstrual Pain

Although the overall time×group interaction did not reach statistical significance (F = 2.693, *p* = 0.083), both groups showed significant reductions in pain intensity following the intervention (F = 101.996, *p* < 0.001). Bonferroni-adjusted pairwise comparisons revealed that, in the MFR group, pain intensity decreased by 2.34 points immediately after the intervention and by 2.00 points at 3 h post-intervention. These reductions exceeded the MCID of 1.65 points, indicating a clinically meaningful effect (Table 2). The main effect of group was not statistically significant (F = 0.704, *p* = 0.409). However, within-group reductions in the MFR group corresponded to a moderate effect immediately after the intervention (*d* = 0.57), suggesting that the immediate reduction was more clinically meaningful than the 3 h effect.

### 3.2. Pressure Pain Threshold at Myofascial Trigger Points

PPTs were assessed at seven myofascial trigger points, where the MFR group consistently demonstrated higher thresholds than the placebo MFR group across the majority of sites. However, no significant main effects of time or time by group interactions were found at any site. Bonferroni-adjusted pairwise comparisons revealed small increases in PPT immediately after and 3 h post-intervention in both groups; however, the between-group differences in these changes were not statistically significant (Supplementary Table S1).

### 3.3. Menstrual Symptoms

Both groups showed significant reductions in menstrual symptoms following the intervention (F = 37.617, *p* < 0.001). Although the overall time×group interaction did not reach statistical significance (F = 3.111, *p* = 0.058), Bonferroni-adjusted pairwise comparisons revealed a significant between-group difference immediately after the intervention, favoring the MFR group (MD = −11.60, 95% CI = −22.37 to −0.83) (Table 3). The main effect of group was not statistically significant (F = 0.073, *p* = 0.789). However, within-group reductions in the MFR group corresponded to a moderate effect immediately after the intervention (*d* = 0.63) and a small-to-moderate effect at 3 h post-intervention (*d* = 0.48), indicating clinically meaningful improvement in menstrual symptoms.

### 3.4. Uterine Artery Hemodynamic Indices

There was a significant time×group interaction for the right uterine artery PI (F = 8.159, *p* = 0.001) and RI (F = 5.249, *p* = 0.011). Bonferroni-adjusted pairwise comparisons revealed significant between-group differences at 3 h post-intervention in the MFR group (PI: MD = −0.97, 95% CI = −1.54 to −0.39; RI: MD = −0.07, 95% CI = −0.11 to −0.02). In the MFR group, PI decreased by 0.69 and RI by 0.05 at 3 h post-intervention (Table 3). The main effects of group were not statistically significant for PI (F = 0.651, *p* = 0.427) or RI (F = 0.769, *p* = 0.389). However, these reductions corresponded to a large effect for PI (*d* = 0.92) and a moderate effect for RI (*d* = 0.70). No significant main effects of time or time×group interactions were observed in the left uterine artery or the arcuate artery. Results for the arcuate artery are not presented, as no significant changes were observed. The arcuate artery is a distal branch of the uterine artery, which may account for its limited sensitivity in detecting hemodynamic changes (Table 3).

## 4. Discussion

### 4.1. Analgesic and Symptom-Related Effects of MFR

This study evaluated the effects of a single session of MFR on pain intensity, menstrual symptoms, and uterine hemodynamic indices in women with PD. Although both groups showed significant reductions in menstrual pain immediately and 3 h post-intervention, no significant group-by-time interaction effects were observed. The reduction in menstrual pain intensity exceeded the MCID of 1.65 in both groups, although no statistically significant interaction effect was found. These findings suggest a potential clinical benefit of MFR, even in the absence of statistically significant between-group differences.

The analgesic effects of MFR may be attributed to physiological mechanisms involving the stimulation of cutaneous and fascial mechanoreceptors and free nerve endings. This stimulation is thought to inhibit sympathetic activity while enhancing parasympathetic tone [36], potentially alleviating uterine muscle spasms, improving local blood flow and metabolic conditions, and interrupting the pain-spasm cycle [37]. Furthermore, the release of fascial adhesions and the facilitation of metabolic waste clearance may support tissue recovery and contribute to pain reduction [20]. Beyond localized effects, MFR has been proposed to modulate viscerosomatic connections along thoracolumbar and sacral segments, potentially impacting autonomic tone and pelvic vascular dynamics in conditions like dysmenorrhea [11]. While the overall protocol was standardized, therapists tailored their responses to each participant’s tissue characteristics and comfort, allowing for subtle adaptations in technique. Such responsiveness may have enhanced parasympathetic activation and vascular effects by promoting relaxation and reducing protective muscle tension.

However, the significant pain reduction observed in both groups necessitates consideration of shared treatment components. In particular, thermotherapy was applied to all participants and is known to relieve muscle tension, improve peripheral circulation, and reduce nociceptor sensitivity, all of which can contribute to analgesic effects [38]. This aligns with previous findings indicating that superficial heat applications at 40–45 °C can alleviate muscular tightness and cramping pain [39]. While the placebo MFR was designed to provide only superficial contact, the possibility of unintended sensory input, such as cutaneous or subcutaneous mechanoreceptor stimulation, cannot be entirely excluded. Given the anatomical continuity between the skin and deeper fascial layers via the retinacula cutis [40]. Therefore, even light touch or warmth from the therapist’s hands during placebo MFR may have modulated sensory processing and nociception, resulting in unintended physiological responses that diminished the between-group contrast. This interpretation is supported by previous studies showing that physical touch, warmth, and therapeutic ritual can evoke placebo effects, particularly in pain-related conditions [41].

Previous studies have also reported that MFR reduces menstrual pain [20,21], which aligns with the present findings. By focusing on a single-session intervention, this trial provides complementary evidence of the short-term effects of MFR in women with PD.

Menstrual symptoms significantly decreased in both groups immediately and 3 h after the intervention. The MDQ-T, used to assess menstrual symptoms, captures autonomic responses, emotional states, and behavioral changes [29]. Improvements in these domains may reflect not only physiological changes but also psychological effects related to the intervention setting. Indeed, verbal suggestion and therapeutic expectancy have been shown to induce autonomic and emotional regulation, producing measurable physiological responses [42].

### 4.2. Vascular Responses to MFR

Notably, significant group by time interactions were observed for the PI and RI of the right uterine artery, with a significant reduction in vascular resistance observed in the MFR group at 3 h post-intervention. Since superficial thermotherapy primarily affects surface tissues and has transient effects, these findings likely reflect a specific physiological response to MFR. Uterine blood flow is regulated by the autonomic nervous system, with sympathetic activation increasing vascular resistance and parasympathetic dominance promoting vasodilation. The observed reduction in uterine arterial resistance following MFR aligns with a hypothesized mechanism involving fascial tension release, autonomic modulation, and improved vascular compliance [11]. The laterality of the effect, limited to the right uterine artery, may reflect anatomical or functional asymmetries in pelvic autonomic innervation [43], a phenomenon previously noted in gynecologic and pelvic pain research. While these changes are promising and align with proposed mechanisms of MFR, further studies are needed to determine whether they persist over time and are specifically attributable to MFR rather than other contributing factors such as residual thermal effects.

### 4.3. Clinical Implications

This study contributes to the existing literature by incorporating objective, quantitative assessments of uterine blood flow using Doppler ultrasonography, an approach that complements traditional self-reported measures of pain and menstrual symptoms. This methodological strength allows for a more comprehensive evaluation of MFR’s physiological impact in women with PD, offering empirical support for its potential to modulate both symptomatic and vascular parameters. These vascular responses, evaluated through Doppler indices, provide individualized physiological insights that may support the integration of MFR into more tailored, patient-centered approaches to dysmenorrhea management. The findings may also support the use of uterine artery resistance indices as surrogate markers of fascial or autonomic interventions in menstrual health.

### 4.4. Limitations and Future Research

Despite these contributions, several limitations should be considered. First, although the placebo MFR was carefully designed to involve only superficial contact, unintended stimulation of cutaneous or subcutaneous mechanoreceptors may have occurred, thereby diminishing the contrast between groups. Second, most participants reported low baseline levels of menstrual pain, which may have limited the sensitivity of the outcomes to detect meaningful between-group differences. Lastly, the use of a single treatment session restricts our ability to conclude long-term or cumulative outcomes. Future studies should explore repeated interventions over extended periods to determine whether MFR exerts dose-dependent or lasting therapeutic effects. Moreover, investigating the integration of MFR with other physical therapy modalities could further enhance the therapeutic approach for PD.

## 5. Conclusions

This study suggests that a single session of MFR may offer clinical benefits for women with primary dysmenorrhea, including pain reduction and improved uterine hemodynamics. The MFR group demonstrated moderate-to-large effect sizes in symptom severity and right uterine artery hemodynamics, reflecting clinically meaningful improvements and suggesting a possible autonomic mechanism. These findings highlight MFR’s potential physiological effects and support its integration as a personalized approach to managing PD. Further research with repeated sessions and larger samples is needed to confirm its long-term efficacy and to further explore its role in individualized therapeutic strategies.

## Figures and Tables

**Table 1:** General characteristics of the study participants (*n* = 34).

	MFR (*n* = 18)	Placebo MFR (*n* = 16)	*p*
Age (years)	22.56 ± 4.64	20.50 ± 1.67	0.093 ^a^
Height (cm)	162.11 ± 5.03	164.04 ± 7.14	0.364 ^a^
Weight (kg)	57.89 ± 6.74	61.38 ± 10.75	0.260 ^a^
BMI (kg/m^2^)	22.04 ± 2.55	22.72 ± 3.06	0.485 ^a^
Menstrual cycle (days)	30.28 ± 4.97	31.25 ± 3.22	0.509 ^a^
Regular exercise			0.642 ^b^
Yes	7 (38.9)	5 (31.3)	
No	11 (61.1)	11 (68.8)	
Menstrual duration			0.479 ^b^
1–3 days	0	1 (6.3)	
4–5 days	10 (55.6)	6 (37.5)	
6–7 days	8 (44.4)	8 (50.0)	
8–9 days	0	1 (6.3)	
Menstrual blood volume (Number of pads per day)			0.549 ^b^
Little bleeding (<5)	4 (22.2)	5 (31.3)	
Moderate bleeding (5–7)	12 (66.7)	11 (68.8)	
Large bleeding (>7)	2 (11.1)	0	
Pain duration			0.604 ^b^
<24 h	5 (27.8)	3 (18.8)	
1–3 days	10 (55.6)	12 (75.0)	
>3 days	3 (16.7)	1 (6.3)	
Taking analgesics			0.173 ^b^
Every month	13 (72.2)	6 (37.5)	
Occasionally	4 (22.2)	7 (43.8)	
No	1 (5.6)	3 (18.8)	
SF-MPQ			
PRI (score)	16.56 ± 6.42	12.50 ± 6.54	0.078 ^a^
Pain over the past week (score)	4.20 ± 1.73	4.18 ± 2.07	0.977 ^a^
PPI			0.818 ^b^
1	3 (16.7)	3 (18.8)	
2	15 (83.3)	12 (75.0)	
3	0	1 (6.3)	

^a^: *p* value is estimated by Independent *t*-test. ^b^: *p* value is estimated by Chi-squared test. Data are presented as mean ± SD or *n* (%). Abbreviations: MFR, Myofascial Release; BMI, Body Mass Index; SF-MPQ, Short Form McGill Pain Questionnaire; PRI, Pain Rating Index; PPI, Present Pain Intensity.

**Table 2:** Changes in menstrual pain over time and between groups (*n* = 34).

Outcome	Groups						Within-Group Difference				Between-Group Difference	
	Pre		Post 1		Post 2		Post 1 Minus Pre		Post 2 Minus Pre		Post 1 Minus Pre	Post 2 Minus Pre
	MFR	Placebo MFR	MFR	Placebo MFR	MFR	Placebo MFR	MFR	Placebo MFR	MFR	Placebo MFR	MFR Minus Placebo MFR	MFR Minus Placebo MFR
NRS	3.86 (0.81)	3.47 (0.80)	1.52 (0.81)	1.79 (0.80)	1.86 (1.10)	1.35 (1.12)	−2.34 (1.14)	−1.69 (1.13)	−2.00 (1.37)	−2.13 (1.38)	−0.65 [−1.32, 0.03]	0.13 [−0.59, 0.84]

All values are adjusted for age, body mass index, pain duration, regular exercise, blood volume, and pain over the past week. Abbreviations: NRS, Numeric Rating Scale.

**Table 3:** Changes in menstrual symptoms and uterine artery hemodynamic indices over time and between groups (*n* = 34).

Outcome	Groups						Within-Group Difference				Between-Group Difference	
	Pre		Post 1		Post 2		Post 1 Minus Pre		Post 2 Minus Pre		Post 1 Minus Pre	Post 2 Minus Pre
	MFR	Placebo MFR	MFR	Placebo MFR	MFR	Placebo MFR	MFR	Placebo MFR	MFR	Placebo MFR	MFR Minus Placebo MFR	MFR Minus Placebo MFR
MDQ-T	36.94 (16.04)	29.07 (16.08)	10.71 (8.82)	14.45 (8.88)	11.16 (10.06)	12.45 (10.08)	−26.23 (18.30)	−14.63 (18.37)	−25.78 (18.93)	−16.63 (18.98)	−11.60 [−22.37, −0.83]	−9.15 [−19.35, 1.05]
UA_L												
PI	3.66 (0.72)	3.47 (0.72)	3.72 (0.89)	3.30 (0.92)	3.14 (0.85)	3.44 (0.84)	0.07 (1.15)	−0.17 (1.17)	−0.51 (1.11)	−0.02 (1.11)	0.24 [−0.29, 0.76]	−0.49 [−1.06, 0.08]
RI	0.98 (0.04)	0.96 (0.04)	0.98 (0.08)	0.93 (0.08)	0.95 (0.08)	0.95 (0.08)	−0.01 (0.09)	−0.03 (0.09)	−0.04 (0.09)	−0.01 (0.09)	0.02 [−0.02, 0.06]	−0.03 [−0.09, 0.03]
UA_R												
PI	3.71 (0.72)	3.16 (0.72)	3.50 (0.93)	3.04 (0.96)	3.01 (0.76)	3.44 (0.76)	−0.21 (1.18)	−0.12 (1.20)	−0.69 (1.05)	0.28 (1.05)	−0.09 [−0.69, 0.51]	−0.97 [−1.54, −0.39]
RI	0.98 (0.04)	0.94 (0.08)	0.95 (0.08)	0.91 (0.08)	0.93 (0.08)	0.96 (0.08)	−0.03 (0.09)	−0.03 (0.11)	−0.05 (0.09)	0.02 (0.11)	−0.002 [−0.05, 0.05]	−0.07 [−0.11, −0.02]

All values are adjusted for age, body mass index, pain duration, regular exercise, blood volume, and pain over the past week. Abbreviations: MDQ-T, Menstrual Distress Questionnaire Form Today; pre, before the intervention; post 1, immediately after intervention; post 2, 3 h post-intervention; MFR, Myofascial Release; UA, Uterine Artery; L, left; R, right; PI, Pulsatility Index; RI, Resistive Index.

## Data Availability

The datasets generated in this study are available from the corresponding author upon request.

## Supplementary Materials

The following supporting information can be downloaded at: https://www.mdpi.com/article/10.3390/medicina61101736/s1, Table S1: Changes in pressure pain threshold at myofascial trigger points over time and between groups (*n* = 34).
