# Supplementary material for: Effect of Myofascial Release on Pain and Uterine Artery Hemodynamic Indices in Women with Primary Dysmenorrhea: A Randomized Controlled Trial
Source: Medicina (Kaunas). 2025 Sep 24;61(10):1736. doi: 10.3390/medicina61101736 (PMC12566312; doi:10.3390/medicina61101736)
Supplement: Supplementary file 1 [file medicina-61-01736-s001.zip › medicina-3818409-supplementary.pdf]

Supplementary Table 1

Table S1. Changes in pressure pain threshold at myofascial trigger points over time and between groups (*n*=34)

MTrP	Groups						Within-group difference				Between-group difference	
	Pre		Post 1		Post 2		Post 1 minus pre		Post 2 minus pre		Post 1 minus pre	Post 2 minus pre
	MFR	placebo MFR	MFR	placebo MFR	MFR	placebo MFR	MFR	placebo MFR	MFR	placebo MFR	MFR minus placebo MFR	MFR minus placebo MFR
Point a	3.32 (1.02)	2.41 (1.00)	3.45 (0.89)	2.44 (0.88)	3.55 (0.89)	2.69 (0.92)	0.13 (1.35)	0.03 (1.33)	0.23 (1.35)	0.28 (1.36)	0.10 [-0.38, 0.58]	-0.05 [-0.57, 0.48]
Point b	3.30 (0.89)	2.56 (0.88)	3.43 (0.89)	2.50 (0.88)	3.57 (1.02)	2.90 (1.04)	0.12 (1.26)	-0.06 (1.24)	0.26 (1.35)	0.34 (1.36)	0.18 [-0.49, 0.85]	-0.08 [-0.80, 0.64]
Point c	3.33 (0.98)	2.51 (1.00)	3.62 (0.68)	2.48 (0.72)	3.48 (0.85)	2.76 (0.88)	0.28 (1.19)	-0.04 (1.23)	0.15 (1.29)	0.25 (1.33)	0.32 [-0.24, 0.89]	-0.10 [-0.63, 0.44]
Point d	3.97 (1.27)	2.84 (1.24)	3.80 (0.85)	2.72 (0.84)	3.84 (1.06)	2.95 (1.04)	0.004 (1.53)	-0.12 (1.50)	0.05 (1.66)	0.11 (1.62)	0.12 [-0.59, 0.84]	-0.06 [-0.66, 0.53]
Point e	3.61 (1.10)	2.50 (1.12)	3.79 (0.72)	2.87 (0.72)	3.74 (1.02)	2.89 (1.00)	0.19 (1.32)	0.37 (1.33)	0.12 (1.50)	0.39 (1.50)	-0.18 [-0.69, 0.33]	-0.27 [-0.74, 0.20]
Point f	3.70 (1.32)	2.89 (1.32)	3.96 (0.85)	3.01 (0.88)	3.86 (0.98)	3.22 (1.00)	0.26 (1.57)	0.11 (1.59)	0.16 (1.64)	0.33 (1.66)	0.15 [-0.55, 0.84]	-0.17 [-0.72, 0.38]
Point g	8.86 (2.55)	8.23 (2.56)	9.15 (2.42)	8.43 (2.40)	9.52 (2.67)	8.71 (2.68)	0.29 (3.51)	0.20 (3.51)	0.65 (3.69)	0.48 (3.71)	0.09 [-0.91, 1.09]	0.17 [-1.07, 1.42]

All values are adjusted for age, body mass index, pain duration, regular exercise, blood volume, pain.

Abbreviations: CI, Confidence Interval; pre, before the intervention; post 1, immediately after intervention; post 2, 3 hours post-intervention; MFR, Myofascial Release; Point a, intersection between the left ASIS and the lateral margin of the rectus abdominis; Point b, intersection between the right ASIS and the lateral margin of the rectus abdominis; Point c, approximately 2 cm medial to the left ASIS; Point d, approximately 2 cm medial to the right ASIS; Point e, left side of the pubic symphysis; Point f, right side of the pubic symphysis; Point g, sacral region (S2–S4)
